# The Associations of Dyadic Coping and Relationship Satisfaction Vary between and within Nations: A 35-Nation Study

**DOI:** 10.3389/fpsyg.2016.01106

**Published:** 2016-08-08

**Authors:** Peter Hilpert, Ashley K. Randall, Piotr Sorokowski, David C. Atkins, Agnieszka Sorokowska, Khodabakhsh Ahmadi, Ahmad M. Alghraibeh, Richmond Aryeetey, Anna Bertoni, Karim Bettache, Marta Błażejewska, Guy Bodenmann, Jessica Borders, Tiago S. Bortolini, Marina Butovskaya, Felipe N. Castro, Hakan Cetinkaya, Diana Cunha, Oana A. David, Anita DeLongis, Fahd A. Dileym, Alejandra D. C. Domínguez Espinosa, Silvia Donato, Daria Dronova, Seda Dural, Maryanne Fisher, Tomasz Frackowiak, Evrim Gulbetekin, Aslıhan Hamamcıoğlu Akkaya, Karolina Hansen, Wallisen T. Hattori, Ivana Hromatko, Raffaella Iafrate, Bawo O. James, Feng Jiang, Charles O. Kimamo, David B. King, Fırat Koç, Amos Laar, Fívia De Araújo Lopes, Rocio Martinez, Norbert Mesko, Natalya Molodovskaya, Khadijeh Moradi, Zahrasadat Motahari, Jean C. Natividade, Joseph Ntayi, Oluyinka Ojedokun, Mohd S. B. Omar-Fauzee, Ike E. Onyishi, Barış Özener, Anna Paluszak, Alda Portugal, Ana P. Relvas, Muhammad Rizwan, Svjetlana Salkičević, Ivan Sarmány-Schuller, Eftychia Stamkou, Stanislava Stoyanova, Denisa Šukolová, Nina Sutresna, Meri Tadinac, Andero Teras, Edna L. Tinoco Ponciano, Ritu Tripathi, Nachiketa Tripathi, Mamta Tripathi, Noa Vilchinsky, Feng Xu, Maria E. Yamamoto, Gyesook Yoo

**Affiliations:** ^1^Department of Psychiatry and Behavioral Sciences, University of WashingtonSeattle, DC, USA; ^2^Department of Psychology, University of ZurichZurich, Switzerland; ^3^Counseling and Counseling Psychology, Arizona State UniversityTempe, AZ, USA; ^4^Institute of Psychology, University of WroclawWroclaw, Poland; ^5^Behavioral Sciences Research Center, Baqiyatallah University of Medical SciencesTehran, Iran; ^6^Department of Psychology, College of Education, King Saud UniversityRiyadh, Saudi Arabia; ^7^School of Public Health, University of GhanaLegon, Ghana; ^8^Department of Psychology, Catholic University of MilanMilan, Italy; ^9^Department of Psychology, The Chinese University of Hong KongHong Kong, China; ^10^Graduate Program in Morphological Sciences, Federal University of Rio de JaneiroRio de Janeiro, Brazil; ^11^Cognitive and Behavioral Neuroscience Unit, D'Or Institute for Research and EducationRio de Janeiro, Brazil; ^12^Institute of Ethnology and Anthropology, Russian Academy of SciencesMoscow, Russia; ^13^Laboratory of Evolution of Human Behavior, Federal University of Rio Grande do NorteNatal, Brazil; ^14^Department of Psychology, Faculty of Languages History and Geography, Ankara UniversityAnkara, Turkey; ^15^Faculty of Psychology and Education Sciences, University of CoimbraCoimbra, Portugal; ^16^Department of Clinical Psychology and Psychotherapy, Babes-Bolyai University Cluj-NapocaCluj-Napoca, Romania; ^17^Department of Psychology, University of British ColumbiaVancouver, BC, Canada; ^18^Department of Psychology, King Saud UniversityRiyadh, Saudi Arabia; ^19^Department of Psychology, Universidad IberoamericanaCiudad de Mexico, Mexico; ^20^Faculty of Arts and Sciences, Izmir University of EconomicsIzmir, Turkey; ^21^Department of Psychology, Saint Mary's UniversityHalifax, NS, Canada; ^22^Department of Psychology, Akdeniz UniversityAntalya, Turkey; ^23^Department of Anthropology, Cumhuriyet UniversitySivas, Turkey; ^24^Faculty of Psychology, University of WarsawWarsaw, Poland; ^25^Department of Public Health, Medical School, Federal University of UberlândiaUberlândia, Brazil; ^26^Department of Psychology, University of ZagrebZagreb, Croatia; ^27^Department of Clinical Services, Federal Neuro-Psychiatric HospitalBenin-City, Nigeria; ^28^Department of Organization and Human Resources Management, Central University of Finance and EconomicsBeijing, China; ^29^Department of Psychology, University of NairobiNairobi, Kenya; ^30^Department of Psychology, Simon Fraser UniversityBurnaby, BC, Canada; ^31^Department of Anatomy, Baskent UniversityAnkara, Turkey; ^32^Department of Social Psychology, University of GranadaGranada, Spain; ^33^Institute of Psychology, University of PécsPécs, Hungary; ^34^Department of Agricultural Extension and Education, Razi UniversityKermanshah, Iran; ^35^Institute of Psychology, University of Science and CultureTehran, Iran; ^36^Department of Psychology, Pontifical Catholic University of Rio de JaneiroRio de Janeiro, Brazil; ^37^Faculty of Computing and Management Science, Makerere University Business SchoolKampala, Uganda; ^38^Department of Pure & Applied Psychology, Adekunle Ajasin UniversityAkungba-Akoko, Nigeria; ^39^School of Education and Modern Languages, Universiti Utara MalaysiaSintok, Malaysia; ^40^Department of Psychology, University of NigeriaNsukka, Nigeria; ^41^Department of Anthropology, Istanbul UniversityIstanbul, Turkey; ^42^Faculty of Arts and Humanities, University of MadeiraFunchal, Portugal; ^43^Institute of Clinical Psychology, University of KarachiKarachi, Pakistan; ^44^Department of Psychological Sciences, Constantine The Philosopher University in NitraNitra, Slovakia; ^45^Department of Social Psychology, University of AmsterdamAmsterdam, Netherlands; ^46^Department of Psychology, South-West University “Neofit Rilski”Blagoevgrad, Bulgaria; ^47^Department of Psychology, Matej Bel University in Banská BystricaBanská Bystrica, Slovakia; ^48^Faculty of Sports and Health Education, Indonesia University of EducationBandung, Indonesia; ^49^Institute of Psychology, University of TartuTartu, Estonia; ^50^Institute of Psychology, University of the State of Rio de JaneiroRio de Janeiro, Brazil; ^51^Organizational Behaviour and Human Resource Management, Indian Institute of Management BangaloreBangalore, India; ^52^Department of Humanities and Social Sciences, Indian Institute of Technology GuwahatiGuwahati, India; ^53^Department of Psychology, Bar-Ilan UniversityRamat-Gan, Israel; ^54^Department of Education for Students, Guangdong Construction PolytechnicGuangdong, China; ^55^Department of Child & Family Studies, Kyung Hee UniversitySeoul, South Korea

**Keywords:** dyadic coping, relationship satisfaction, culture, multilevel modeling, gender differences

## Abstract

**Objective:** Theories about how couples help each other to cope with stress, such as the systemic transactional model of dyadic coping, suggest that the cultural context in which couples live influences how their coping behavior affects their relationship satisfaction. In contrast to the theoretical assumptions, a recent meta-analysis provides evidence that neither culture, nor gender, influences the association between dyadic coping and relationship satisfaction, at least based on their samples of couples living in North America and West Europe. Thus, it is an open questions whether the theoretical assumptions of cultural influences are false or whether cultural influences on couple behavior just occur in cultures outside of the Western world.

**Method:** In order to examine the cultural influence, using a sample of married individuals (*N* = 7973) from 35 nations, we used multilevel modeling to test whether the positive association between dyadic coping and relationship satisfaction varies across nations and whether gender might moderate the association.

**Results:** Results reveal that the association between dyadic coping and relationship satisfaction varies between nations. In addition, results show that in some nations the association is higher for men and in other nations it is higher for women.

**Conclusions:** Cultural and gender differences across the globe influence how couples' coping behavior affects relationship outcomes. This crucial finding indicates that couple relationship education programs and interventions need to be culturally adapted, as skill trainings such as dyadic coping lead to differential effects on relationship satisfaction based on the culture in which couples live.

## Introduction

Stress that spills over into one's intimate relationship (Repetti, 1989) can increase negative behavior between partners (Repetti, 1989; Schulz et al., 2004), which in turn can negatively affect relationship outcomes, such as satisfaction (Karney and Bradbury, 1995; Randall and Bodenmann, 2016). This negative stress spillover process may, however, be mitigated if couples help each other cope with the experienced stress (i.e., dyadic coping). Although theoretical assumptions, such as the systematic-transactional model of stress and dyadic coping (Bodenmann, 2005), suggest that the association between coping behavior and relationship satisfaction is determined by cultural influences (e.g., gender roles), findings from a recent meta-analysis shows that this association is stable across nations and gender (Falconier et al., 2015). Despite the significant findings, the samples used in the meta-analysis nearly exclusively relied on couples living in Western culture (Falconier et al., 2015), which leaves an unanswered question about how culture may affect the association between dyadic coping and relationship satisfaction. The goal of the current paper was to examine the cultural influence in dyadic coping processes based on 7973 married individuals across 35 nations.

## Stress and stress-spill-over processes

People all over the globe experience a variety of stressors, which can have negative effects on their everyday life and well-being (Thoits, 2010). Most people experience stress stemming from work, finances, children, illness, and disagreements with others (Randall and Bodenmann, 2016), whereas some people face additional stressors such as poverty, high crime rates, epidemics, hunger crises, and wars (Hilpert and Kimamo, 2016). Research shows that stress can have detrimental effects on an individual's mental (Thoits, 2010) and physical well-being (Larzelere and Jones, 2008).

Importantly, stress does not just affect the individual; rather, it can be considered a dyadic construct wherein it impacts both members of a dyad (Randall and Bodenmann, 2009, 2016). There is ample evidence to suggest that stress spills over into one's intimate relationship (Repetti, 1989; Neff and Karney, 2009; Hilpert et al., 2015). In more detail, experiencing a stressful situation may increase an individual's stress experience, which in turn may increase how they interact with their partner (e.g., more conflict behavior; Bodenmann et al., 2007). Findings on stress spillover show that on days when individual's experience higher levels of work stress, women express more anger and men withdraw more (Schulz et al., 2004).

Evidence shows that one-third of all stable couples are unhappy with their relationship (Whisman et al., 2008), and 30–50% of marriages in North America and Europe end in divorce (Bramlett and Mosher, 2002; Eurostat, 2007). Most theories suggest that the cause of relationship problems lies within the interdependent interactions between partners (Thibaut and Kelley, 1959), based on their problematic personality traits (e.g., neuroticism; Story and Bradbury, 2004), or lack of communication skills (Burleson and Denton, 1997). However, the stress spillover phenomenon in couples provides an explanation for why even “happy” and “functional” relationships may eventually fail over time as living in stressful environments may erode communication skills and functional behaviors (Bodenmann, 2005).

## Coping processes in couples: a brief theoretical overview

According to the Systemic-Transactional Model (STM; Bodenmann, 1995, 1997, 2005), the stress-coping process is an interdependent process between two members of a dyad. The STM suggests that stress experienced by one partner affects the other partner as well because stress is communicated either verbally or non-verbally (Bodenmann, 1995). Most importantly, the STM suggests that the negative impact of stress on intimate relationships can be buffered if partners help each other to positively cope with stress (hereafter dyadic coping; Randall and Bodenmann, 2009, 2016).

Partners can engage in a number of dyadic coping behaviors, such as supportive, common, delegated, and even negative dyadic coping behaviors. For example, showing empathic understanding and expressing solidarity when the partner is stressed (e.g., work stress) is defined as *supportive dyadic coping*. If both partners experience a common stressor (e.g., parenting, financial problems), the role of support seeking and providing is more symmetrical as both are stressed and can try to help each other, which is specified as *common dyadic coping*. In the current study, the two subscales supportive and common dyadic coping were aggregated (Bodenmann, 2008).

Conceptually, it is predicted that engaging in positive dyadic coping has a positive impact on relationship satisfaction (Cutrona, 1996). Dyadic coping not only prevents an increase in negative behavior between partners but rather, supporting one's partner during times of distress can have a positive effect on relationship functioning. Specifically, partners may feel understood and cared for, which has been found to be associated with greater intimacy, trust, and relationship satisfaction (Cohen and Wills, 1985; Hilpert et al., 2013, 2015).

It is reasonable to assume that the association between dyadic coping and relationship satisfaction varies between men and women. According to evolutionary perspective and attachment theory, women have a higher investment in parenting and tend to be the primary caregiver, which provides an advantage to providing support (Bolby, 1969). Eagly and Wood (1999) suggest that gender difference in behavior within a romantic relationship can occur based on evolved predispositions or based on role models men and women have within in the social system. Cutrona (1996) argues that the quality of support is higher in women than in men, indicating that men benefit more in comparison with women.

## How culture influences the coping processes in couples

Generally speaking, culture influences people's behavior (Markus and Kitayama, 1991). According to several conceptual models of how couples cope with stress (Revenson, 1990, 2003; Bodenmann, 1995; Berg and Upchurch, 2007; Falconier et al., 2016), culture is seen as a contextual factor that influences how couples help each other to cope (Falconier et al., 2016), but little is known about how culture affects couples' coping behaviors. We summarize four cultural constructs that may affect partner's coping behavior: individualism/collectivism, family situation (nuclear/extended), gender roles, and communication (Falconier et al., 2016).

### Individualism-collectivism

People in individualistic cultures strive to achieve their individual goals, whereas people in collectivistic cultures work toward the goals of their family or larger social group (Triandis, 2001; Oyserman et al., 2002; Shek, 2006). Nations and continents such as North America, Europe, Australia, and South Africa represent individualistic cultures, whereas Asia, Africa, and South America represent collectivist cultures (Hofstede, 1980; Triandis, 1988). Prior research has shown that Asians and Asian Americans seek less support from close others in comparison with Americas when undergoing stressful experiences because they are concerned that mobilizing support might disturb the harmony with close others (Taylor et al., 2004; Kim et al., 2006). Thus, we propose that when partners who identify with a collectivistic culture experience stress, they might provide less explicit support and the provided support might be less effective (Kuo, 2013).

### Family situation

Cultures differ in the extent to which couples live in nuclear families (i.e., spouses and children only) or in extended families (i.e., together with grandparents and other relatives; Georgas et al., 2001). Living in nuclear families is more common in Western, individualistic, nations, which means that the partner may be, for most, the primary support provider (Bodenmann, 2000). The coping dynamics in couples in extended families might be very different as their family network is larger and more people can provide support (Hilpert and Kimamo, 2016).

### Gender roles

Gender roles reflect cultural norms that which behaviors are appropriate for men and women in a relationship. Gender roles for women are different for women in Western, individualistic, cultures where they can participate more freely in the society, whereas the gender roles might be very different if a woman lives in Africa or the Middle East (Giuliano and Nunn, 2013). In a society where men and women share egalitarian gender roles, the coping process can be congruent and consistent as men and women can equally seek and provide support to maintain their relationship (Bodenmann, 2005). However, in a society where the perception of men and women' roles are asymmetric, the process is more one-sided. An example of this can be seen in China, where women are in a submissive role and are seen more as support providers, whereas men are support receivers. Nevertheless, these roles are changing in China, as most women are employed and demand the equal sharing of both family and work affairs (Shek, 2006; Xu et al., 2016).

### Communication

People in individualistic culture are thought to be more explicit in their communication and depend less on contextual cues, such as gestures (Shibusawa, 2005), whereas collectivist cultures rely more on indirect communication and contextual cues (Gao et al., 1998). The main explanation for this phenomenon is that harmony in Eastern countries is highly favored which may make them suppress their ways of communication (Gao et al., 1998). Based on these styles of communication, one could infer that the coping process between partners would look very different depending on cultural context.

A recent meta-analysis by Falconier and colleagues (Falconier et al., 2015) examining all articles published on dyadic coping until 2013 showed that dyadic coping behavior is strongly associated with relationship satisfaction (*r* = 0.45; *p* < 0.001), using a sample of 17,856 participants across 13 Western cultures. Furthermore, these results show that the effect between dyadic coping and relationship satisfaction is stable across Western nations.

Despite the strong theoretical assumptions that the coping process for men and women should differ (Revenson, 2003; Bodenmann, 2005; Berg and Upchurch, 2007), this claim has hardly been empirically supported. For example, no difference could be found between women's and men's coping processes (Zwicker and DeLongis, 2010; Hilpert et al., 2013) and in daily diary studies men are equally skilled in providing support as are women (Neff and Karney, 2005; Iida et al., 2008). Finally, the results of the meta-analysis (Falconier et al., 2015) did not provide evidence for gender differences in couples' dyadic coping behavior.

However, all these findings are based on Western couples. With the exception of one study from Indonesia (*k* = 1), all other studies (*k* = 71) from the meta-analysis were based on couples living in a Western culture. As noted above, grounded in cross-cultural research on family situation, gender roles, and communication, it could be suggested that cultural aspects related to these domains may influence the coping processes in couples between nations and across the globe, but there is no empirical evidence to support this to date. This is a critical question, as couple's relationship education programs and interventions depend on the assumption that the knowledge gained, for example in regards to stress communication, will lead to a reliable increase in relationship satisfaction. However, if we find that effects of dyadic coping depend on the couples' culture, the results of this study could have important implications for cultural adaptations in relationship education programs.

## Current study

Dyadic coping has been shown to have important implications for individual well-being and relationship outcomes (Bodenmann et al., 2011). However, nearly all studies examining the associations between dyadic coping and relational outcomes have been conducted with Western samples, which leaves gaps in understanding how culture may influence couples' coping processes across the globe. This study addresses this gap and examines the association between dyadic coping and relationship satisfaction in 35 nations, representing nations from North America (U.S., Canada), Europe (Bulgaria, Croatia, Germany, Greece, Hungary, Italy, Poland, Portugal, Romania, Slovakia, Spain, Switzerland, U.K.), former Soviet republic (Kazakhstan, Russia, Estonia), Asia (China, Hong-Kong, Indonesia, India, Malaysia, Pakistan, South Korea), Middle East (Iran, Israel, Saudi-Arabia, Turkey), South America (Brazil, Mexico), and Africa (Ghana, Kenia, Nigeria, Uganda).

## Hypotheses

Based on the above-mentioned empirical findings, we hypothesize that dyadic coping will be significantly associated with relationship satisfaction across all nations (H1a). Based on theoretical assumptions that culture affects the coping process in couples (Bodenmann, 1995; Revenson, 2003; Berg and Upchurch, 2007), we further hypothesize that there will be significant variability between nations in the association between dyadic coping and relationship satisfaction (H1b). Additionally, we hypothesize that gender will significantly moderate the association between dyadic coping and relationship satisfaction across all nations (H2a). Based on the variability of gender role difference across the globe (egalitarian, asymmetric), we further hypothesize that the association will be higher for women in some nations but not in others (H2b).

## Method

### Sample

Data for this study were collected from 35 nations with a total of 7973 participants (3584 men; 4349 women). The average sample size per nation was 228 participants, and only one sample was smaller than 90 participants (Romania, *n* = 56). Participants had to be at least 18 years of age and had to be married in order to participate.

Participants age ranged from 18 to 88 years old (men: *M* = 40.8, *SD* = 11.8, range: 18–88; women: *M* = 40.5, *SD* = 11.5, range: 18–86). Average length of marriage was 14.7 years (men: *M* = 14.2, *SD* = 11.6, range: 0–70; women: *M* = 15.0, *SD* = 11.9, range: 0–65), and participants had, on average, 1.8 children (*SD* = 1.4, range: 1–9). Overall, our sample was educated with 53% reporting having a bachelor's degree or higher, 14% had a high school or technical college, 5% completed secondary school, and 1% had finished primary schooling.

### Design

Researchers across the globe were invited to collect data in their nation for a study on how demographic variables predict relationship satisfaction (see authors, under review). In total, researchers from 35 nations agreed to contribute to the study and collected 46 samples (i.e., in some countries more than one sample was collected). Recruitment methods were chosen by the research team in each country, but contributors were requested to assess a heterogeneous sample according to age, relationship duration, education, and income. Forty-three samples were collected using an in-person, paper-pencil approach, and three samples were collected online (Canada, Israel, and USA). Collaborators using the paper-pencil approach assessed participant's data in the presence of the investigator in order to limit external influences. Note that only married individuals were recruited. Thus, data came from married individuals but not from both partners in the dyad. All participants were notified that their data would be anonymized and kept confidential and that they could discontinue the study at any time without facing any problem. Informed consent was obtained for all participants in the current study. The cross-cultural study was approved by the ethical commission at the University of Wroclaw. If the general ethical approval was not enough in a specific nation, collaborator got ethical approval from their University (i.e., University of British Columbia, University of Arizona, and Bar-Ilan University). Participants did not get any incentive for their participation.

All measures were administered in English. In all non-English speaking countries, the questionnaire was translated to native languages by research teams fluent in English using a standardized back-translation procedure (Sireci, 2006). The collaborators translated the measures into the native language of the participants, and bilingual persons back-translated the measures into English. Differences between the original English version and the back-translation were discussed, and consensus agreements were made on the most appropriate translation. Problems with the translation were discussed with the primary research team, but no problems were reported. This approach allowed us to avoid biases commonly found in cross-cultural research (Van de Vijver and Leung, 1997).

## Measures

### Socio-demographics

Participants reported basic demographic factors such as gender, age, marriage duration, number of children, and education. We included individualism-collectivism dimension of Hofstede (Hofstede, 2001; Hofstede et al., 2010) and capita nominal gross domestic product (GDP) per country as a control variables.

### Dyadic coping

A short four-item version of the English translated Dyadic Coping Inventory (DCI; Bodenmann, 2008; Randall et al., 2015) was used to assess perceived partners' positive dyadic coping behaviors with two subscales, each with two items: supportive and common dyadic coping. Respondents were asked to evaluate how often their partner provides supportive dyadic coping during stressful times (“*My partner shows empathy and understanding when I need it*;” “*When I am stressed, my partner listens to me and gives me the opportunity to communicate what really bothers me.”*) and how often they provide common dyadic coping when both are stressed (“*When we are stressed, we help one another to put the problem in perspective and see it in a new light*;” “*When we are stressed, we do something together, are affectionate to each other and cope jointly”*) on a 5-point scale (1 = *never* to 5 = *very often*). In this study, α was 0.86 or higher across all samples and across both genders.

### Marital satisfaction

We used the love style subscale of the Marriage and Relationships Questionnaire (Russel and Wells, 1993) to assess relationship satisfaction. Participants evaluated their relationship satisfaction with nine items (“*Do you love your husband/wife?;” “Do you enjoy your husband's/wife's company?”*) on a five-point scale (1 = *no*; 2 = *rather no*; 3 = *neither yes nor no*; 4 = *rather yes*; 5 = *yes*). This subscale has been tested for cross-cultural use and showed good psychometric characteristics in many cross-cultural studies (Lucas et al., 2004, 2008; Weisfeld et al., 2011). In the current study, internal consistency (Cronbach alpha) was 0.91 across all samples and both genders.

### Statistical analyses

#### Hypothesis 1

Our hypotheses focus on the overall associations of dyadic coping and relationship satisfaction (i.e., average association across all nations) as well as nation-to-nation variability in these associations. As such, in the current sample, individuals are nested within nations. People from a specific nation share a common, cultural environment, and the shared environment within a country can be separated from the contribution of each individual. Using multilevel between-within models (Raudenbush and Bryk, 2002) allowed us to model: (i) the effect of dyadic coping across nations (between-nation variability), and (ii) the association of dyadic coping with relationship satisfaction between-persons within a nation (within-nation variability; Eid and Lischetzke, 2013).

In order to analyze between- and within-nation associations, we separated the dyadic coping values of each person into two components: (1) a nation-level mean of dyadic coping (${\bar{DC}}_{\text{j}}$), and (2) the difference between each individual's dyadic coping from their nation-level mean (*DC*_ij_ –${\bar{DC}}_{\text{j}}$), where *i* indexes individuals and *j* indexes countries. Because samples could vary between nations for a variety of reasons (e.g., beyond cultural differences in dyadic coping or marital satisfaction), we aimed to include a set of control variables in the analyses, specifically gender, age, marriage duration, number of children, education, individualism-collectivism, and GDP. In order to find the best fitting model, we followed Zuur et al. (2009) suggestion to use a top-down approach to identify the optimal random and fixed structure. To find the optimal random structure, we included all fixed effects and sequentially compared models included one random effect at the time. Likelihood ratio test showed that a model including random intercepts and a random slope for *DC*_ij_ –${\bar{DC}}_{\text{j}}$ (*p* < 0.001) provided the best model fit. To find the optimal fixed structure, we included all fixed effects and sequentially compared models excluding non-significant effects using deviance tests. Model comparison showed that marital duration, number of children, and individualism-collectivism did not contribut significantly to the model and were therefore excluded from the final model.

To examine how dyadic coping predicts relationship satisfaction across all nations (H1a) and within each nation (H1b), we used the following baseline model:

(1) $$\begin{array}{l} \begin{array}{l} \text{Relationship}\\ {\text{Satisfaction}}_{\text{ij}} \end{array}=\beta_{0}+\beta_{1}({\bar{DC}}_{\text{j}})+\beta_{2}(DC_{\text{ij}}-{\bar{DC}}_{\text{j}})\\ \;+\beta_{3}({\text{gender}}_{\text{ij}})+\beta_{4}({\text{age}}_{\text{ij}})\\ \;+\beta_{5}({\text{education}}_{\text{ij}})\\ \;+\beta_{6}{\text{GDP}}_{\text{j}}+{\text{u}}_{0\text{j}}+{\text{u}}_{1\text{j}}(DC_{\text{ij}}-{\bar{DC}}_{\text{j}})+{\text{r}}_{\text{ij}} \end{array}$$

In Equation 1 the β_1_ coefficient captures the between-nations association of dyadic coping and relationship satisfaction, whereas β_2_ captures the average within-nations association, controlling for mean differences in nations as well as the other included covariates. We predicted relationship satisfaction for an individual in a given nation by the intercept β_0_ (i.e., the predicted value of relationship satisfaction, if all predictors are zero), by the slope of ${\bar{DC}}_{\text{j}}$ (β_1_), the slope of DC_ij_ –${\bar{DC}}_{\text{j}}$ (β_2_), the slope of all control variables (β_3_ to β_6_), u_0*j*_ represents the random intercepts, u_1j_ indicates the random slope for dyadic coping (*DC*_*ij*_ –${\bar{DC}}_{j}$), and the residuum *r*_ij_ represents the residual for person *i* in nation *j* (i.e., the individual deviation of the observed value from the predicted value). Significant effects for these two parameters (β_1_ and β_2_) would support our first hypothesis (H1a).

Furthermore, Hypothesis 1 predicts that the association between dyadic coping and relationship satisfaction is significantly different between nations. An omnibus test of nation-to-nation variability is provided by the variance term for the slope of within-nation dyadic coping (u_1j_). Findings showing significant variance term for the slope u_1*j*_(*DC*_ij_ –${\bar{DC}}_{\text{j}}$) would support H1b. Beyond this, we did not have nation-specific hypotheses (e.g., nation A should have a stronger association than nation B). Moreover, pair-wise comparisons involving 35 nations could yield a total of 595 comparisons. To overcome this problem, Goldstein and Healy (1995) present a method to compare a group of means graphically, via specially constructed confidence intervals. Specifically, the confidence intervals are adjusted to be 83.5% intervals (±1.39σ), and non-overlap of the confidence intervals suggests significant differences in the mean between nations.

#### Hypothesis 2

We hypothesize that the association between dyadic coping and relationship satisfaction differs for husbands and wives across all nations (H2a). In addition and based on the cultural differences in gender roles, we hypothesize that the association between dyadic coping and relationship satisfaction might vary across nations and could be higher for women in some cultures in comparison with the effect for men in their culture (H2b). These hypotheses were tested by using the baseline model (see hypothesis 1), but including two interaction terms: (i) interaction between gender and nation-level mean DCI (${\bar{DC}}_{\text{j}}$) and (ii) interaction between gender and the difference between each individual's DCI from nation-level mean (*DC*_ij_ –${\bar{DC}}_{\text{j}}$). In order to test whether the interactions were significant, we used again the Goldstein and Healy (Goldstein and Healy, 1995) method to compare a group of means graphically, via specially constructed confidence intervals. In this case, the confidence intervals are adjusted to be 95% intervals (±1.96σ), and non-overlap of the confidence intervals with zero suggests significant interaction.

We used R Version 3.0.2 (The R Project for Statistical Computing)^1^ to compute descriptive statistics and the lme4 package in R for multilevel modeling (Bates et al., 2015).

## Results

### Descriptive statistics

Table 1 shows means and standard deviations for all study variables between and within nations. On average, the frequency of positive dyadic coping behavior (*M*_Average_ = 3.69, range 1–5) and the level of relationship satisfaction (*M*_Average_ = 4.50, range 1–5) were relatively high. Results of random intercept model for dyadic coping and marital satisfaction model^2^ indicated significant differences in how often dyadic coping behavior is provided and in how satisfied people are with their marriage between nations. Means of the control variables show that our participants were on average 40.7 years old (*SD* = 11.6), were married for 14.7 years (*SD* = 11.8), had 1.8 children (*SD* = 1.4), had an education level of 4.3 (4 = high school, 5 = bachelor degree; *SD* = 0.9), and an average GDP of $22,282 per annum^3^.

**Table 1:** **Mean and standard deviation for all study variables across all nations and for each nation**.

	***N*_Men_**	***N*_Women_**	***N*_Total_**	**Dyadic coping**		**Marital satisfaction**		**Age**	**Marriage Duration**	**Number of Children**	**Education**	**GDP**
				***M* (*SD*)**	**α**	***M* (*SD*)**	**α**	***M* (*SD*)**	***M* (*SD*)**	***M* (*SD*)**	***M* (*SD*)**	**$**
Average across all nations	3582	4347	7973	3.69 (0.95)	0.86	4.50 (0.64)	0.92	40.7 (11.6)	14.7 (11.8)	1.8 (1.4)	4.3 (0.9)	22,282
**NORTH AMERICA AND WEST EUROPE**												
Canada	68	212	280	3.78 (0.89)	0.88	4.54 (0.68)	0.94	40.1 (13.0)	13.8 (13.0)	1.5 (1.6)	4.6 (0.5)	50,169
Germany	44	60	104	3.79 (0.78)	0.83	4.59 (0.61)	0.94	47.5 (12.5)	17.6 (15.2)	1.7 (1.0)	4.2 (1.0)	47,966
Italy	127	195	322	3.67 (0.87)	0.84	4.64 (0.42)	0.85	48.4 (11.1)	24.6 (11.6)	1.7 (0.9)	4.0 (0.9)	35,812
Portugal	102	183	298	3.70 (0.89)	0.84	4.56 (0.74)	0.96	46.2 (11.2)	21.1 (12.4)	1.6 (0.8)	3.8 (1.0)	22,122
Spain	94	108	202	3.67 (0.94)	0.89	4.57 (0.56)	0.91	47.1 (9.4)	19.4 (10.2)	1.7 (0.9)	3.8 (1.1)	29,861
Switzerland	112	76	188	3.66 (0.81)	0.87	4.59 (0.53)	0.91	48.7 (12.9)	21.1 (13.2)	2.0 (1.2)	4.4 (0.6)	85,374
U.K.	42	58	100	3.89 (0.84)	0.86	4.63 (0.47)	0.92	45.0 (11.6)	19.4 (13.1)	1.7 (1.4)	4.3 (0.7)	46,461
United States of America	86	153	239	3.96 (0.95)	0.90	4.68 (0.54)	0.93	36.8 (12.3)	9.3 (10.4)	1.6 (1.5)	4.6 (0.6)	54,306
**EAST EUROPE**												
Bulgaria	63	39	102	3.92 (0.42)	0.61	3.96 (0.62)	0.92	38.4 (9.0)	8.8 (96.6)	1.1 (0.5)	4.7 (0.8)	7876
Croatia	306	315	621	3.63 (0.89)	0.89	4.44 (0.57)	0.90	44.8 (11.7)	18.2 (11.9)	1.7 (1.1)	4.0 (1.0)	13,425
Greece	44	50	97	3.96 (0.91)	0.85	4.51 (0.66)	0.93	38.7 (9.0)	11.5 (9.8)	1.5 (1.0)	4.2 (0.8)	43,430
Hungary	76	161	237	3.45 (0.80)	0.68	4.43 (0.65)	0.93	37.8 (19.6)	12.6 (9.5)	1.6 (1.0)	4.1 (0.9)	13,989
Poland	166	278	447	3.67 (0.99)	0.90	4.46 (0.69)	0.94	40.6 (11.7)	16.4 (12.0)	1.8 (1.2)	4.4 (0.7)	14,111
Romania	8	48	56	3.72 (1.06)	0.88	4.30 (0.95)	0.95	35.0 (6.7)	8.0 (6.6)	0.9 (0.8)	4.9 (0.5)	10,129
Slovakia	77	157	234	3.58 (0.93)	0.86	4.28 (0.78)	0.93	42.8 (11.8)	18.3 (11.9)	1.8 (1.0)	4.5 (0.6)	23,954
**FORMER SOVIET COUNTRIES**												
Estonia	50	98	151	3.68 (0.87)	0.90	4.51 (0.58)	0.92	42.9 (12.3)	17.1 (12.6)	2.0 (1.1)	4.5 (0.8)	20,122
Kazakhstan	60	60	120	4.00 (0.76)	0.84	4.77 (0.30)	0.76	37.0 (8.2)	13.0 (7.4)	1.9 (0.6)	4.3 (1.0)	12,436
Russia	121	104	225	3.75 (0.60)	0.83	4.50 (0.56)	0.88	38.6 (13.9)	13.8 (13.2)	1.0 (0.8)	4.5 (0.9)	12,972
**ASIA**												
China	47	72	119	3.54 (0.78)	0.84	4.51 (0.59)	0.88	33.1 (6.4)	7.6 (6.7)	1.0 (0.5)	4.5 (1.0)	7617
Hong Kong	54	40	100	3.29 (0.92)	0.91	4.03 (0.91)	0.95	47.1 (10.0)	20.4 (10.5)	1.5 (1.1)	3.9 (1.0)	40,252
India	135	164	299	4.10 (0.97)	0.89	4.77 (0.38)	0.87	34.1 (8.0)	7.6 (7.4)	1.0 (0.8)	4.9 (0.3)	1586
Indonesia	26	67	93	3.74 (0.88)	0.83	4.61 (0.63)	0.92	41.7 (9.9)	15.7 (11.2)	2.0 (1.0)	4.5 (0.9)	3492
Iran	263	345	609	3.34 (1.19)	0.91	4.11 (0.80)	0.88	38.8 (10.9)	15.3 (11.1)	2.1 (2.2)	3.7 (1.1)	5443
Malaysia	49	50	99	4.12 (0.71)	0.83	4.86 (0.35)	0.93	40.0 (8.9)	13.5 (9.2)	2.9 (2.0)	4.5 (0.7)	10,933
Pakistan	58	71	133	3.65 (1.05)	0.90	4.53 (0.63)	0.93	35.9 (10.2)	10.4 (9.7)	1.9 (1.4)	4.8 (0.6)	1561
South Korea	50	50	100	3.52 (0.83)	0.89	4.39 (0.55)	0.91	41.8 (7.7)	15.1 (8.2)	1.7 (0.8)	4.4 (0.6)	28,166
**MIDDLE EAST**												
Israel	75	165	240	3.81 (0.78)	0.81	4.45 (0.69)	0.92	43.0 (12.3)	16.0 (13.5)	2.4 (1.4)	4.9 (0.4)	38,261
Saudi Arabia	87	112	199	3.43 (0.86)	0.72	3.93 (0.63)	0.79	36.2 (8.3)	12.3 (8.5)	2.8 (1.7)	4.6 (0.8)	24,362
Turkey	239	154	393	3.62 (0.95)	0.85	4.59 (0.54)	0.94	42.8 (13.6)	16.7 (13.8)	1.8 (1.2)	4.1 (1.1)	10,299
**AFRICA**												
Ghana	53	51	104	3.81 (0.93)	0.86	4.70 (0.48)	0.90	40.4 (9.5)	12.0 (9.6)	2.5 (1.6)	4.3 (1.1)	1388
Kenya	47	47	94	3.78 (1.07)	0.85	4.67 (0.55)	0.93	32.3 (7.3)	7.6 (6.1)	1.8 (1.2)	4.4 (1.0)	1358
Nigeria	304	298	610	3.86 (1.04)	0.88	4.72 (0.45)	0.88	39.0 (9.1)	10.3 (8.7)	2.5 (1.8)	4.3 (0.9)	3203
Uganda	62	36	100	3.59 (0.47)	0.87	4.47 (0.59)	0.89	34.9 (10.3)	8.2 (8.2)	2.9 (2.1)	4.8 (1.0)	727
**MIDDLE AND SOUTH AMERICA**												
Brazil	304	181	485	3.64 (0.89)	0.84	4.66 (0.50)	0.88	36.5 (10.3)	10.6 (9.9)	1.1 (1.0)	4.6 (0.7)	11,387
Mexico	83	89	173	3.71 (0.98)	0.87	4.65 (0.65)	0.91	39.0 (11.4)	11.7 (10.0)	1.6 (1.2)	4.2 (1.1)	10,326

*GDP = gross domestic product in 2015. Because a few people did not report their gender, the total number of participants differs from the sum of male and female participants in some nations*.

#### Hypothesis 1a

**Figure 1:**
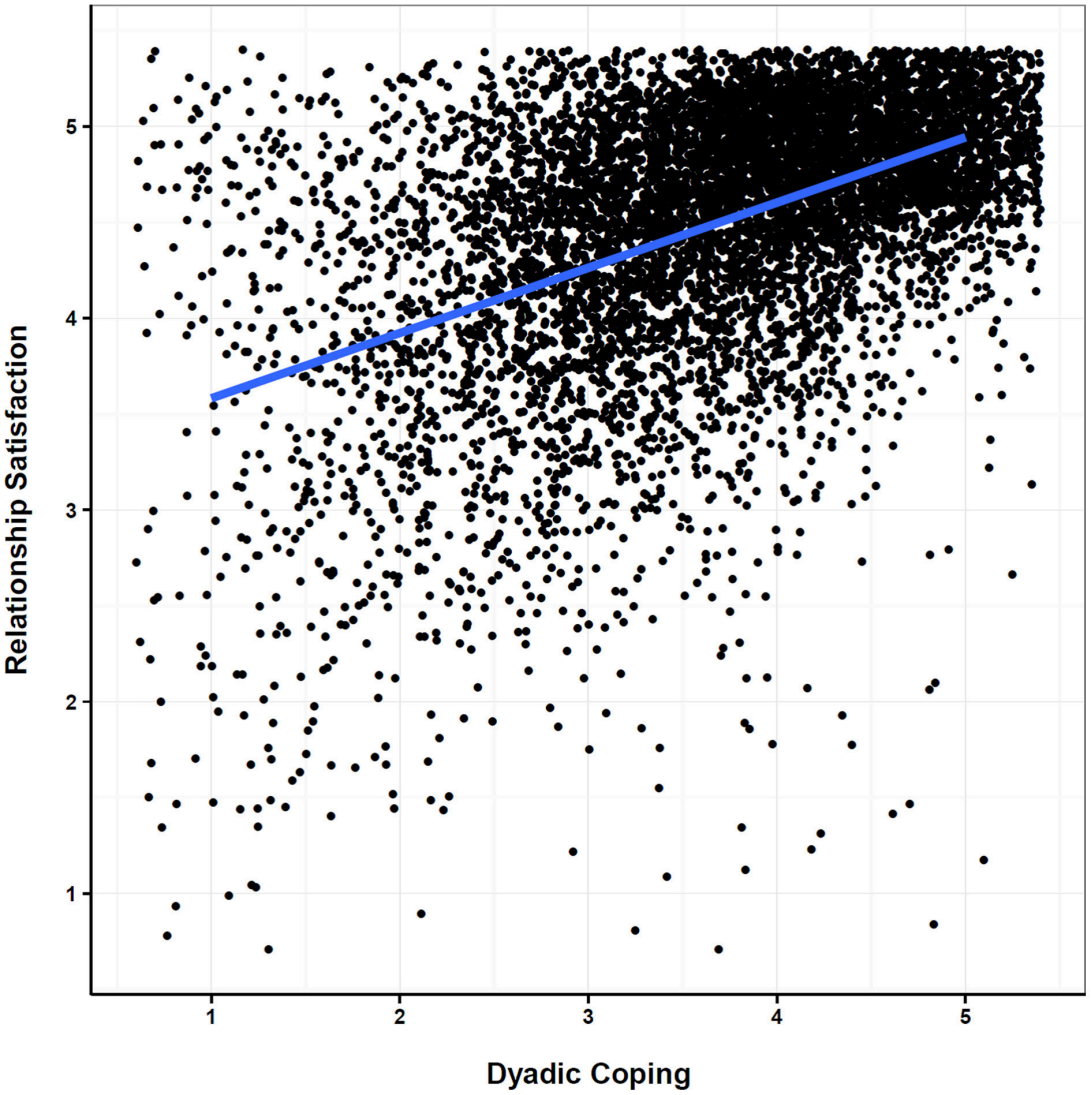
**Scatterplot between dyadic coping and relationship satisfaction across all individuals including an average linear fitting line**.

We hypothesized significant associations between dyadic coping and marital satisfaction across all nations (H1a). The association between dyadic coping and marital satisfaction across all nations is depicted in a scatterplot for all participants with an average linear fitting line (Figure 1). Visual inspection supports a positive association. Fixed effects (i.e., average effect across all nations) of the multilevel analysis are displayed in the upper panel of Table 2. Results show that dyadic coping (${\bar{DC}}_{\text{j}}$) significantly predicted marital satisfaction (β = 0.59; *p* < 0.000), indicating that in nations with higher average dyadic coping scores, couples are on average more satisfied with their marriage in comparison with nations where individuals report less coping behavior from their partner. We further tested for the average within-nation variability of dyadic coping. Results show that couples who perceive more support from their partner in comparison with other couples in the same nation were generally more satisfied with their marriage (β = 0.35; *p* < 0.000). Finally, gender, age, education, and GDP significantly predict marital satisfaction, whereas marriage duration and number of children were excluded from the final model as they did not predict marital satisfaction.

**Table 2:** **Parameter estimates for multilevel model**.

**Fixed Effects (intercept, slope)**	**Estimate**	***SE***	***t***	***p***
Intercept	2.37	0.45	5.4	< 0.000
Dyadic Coping (${\bar{DC}}_{j}$)	0.59	0.12	5.0	< 0.000
Dyadic Coping (*DC_ij_* −${\bar{DC}}_{j}$)	0.35	0.03	13.5	< 0.000
**CONTROL VARIABLES**				
Gender	−0.04	0.01	−3.0	0.002
Age	−0.01	0.01	−5.6	< 0.000
Education	0.02	0.01	3.8	< 0.000
GDP	0.01	0.01	2.3	0.033
**Random Effects ([co-]variances)**	**Slopes**	**Intercepts**	***p***	
**NORTH AMERICA AND WEST EUROPE**				
Canada	0.54	2.25	0.000	
Germany	0.43	2.37	0.000	
Great Britain	0.29	2.37	0.000	
Italy	0.25	2.54	0.000	
Portugal	0.23	2.38	0.000	
Spain	0.35	2.48	0.000	
Switzerland	0.31	2.37	0.000	
United States of America	0.36	2.31	0.000	
**EAST EUROPE**				
Bulgaria	0.78	1.76	0.000	
Croatia	0.40	2.40	0.000	
Greece	0.47	2.21	0.000	
Hungary	0.44	2.46	0.000	
Poland	0.43	2.38	0.000	
Romania	0.56	2.17	0.000	
Slovakia	0.55	2.23	0.000	
**FORMER SOVIET COUNTRIES**				
Estonia	0.38	2.41	0.000	
Kazakhstan	0.25	2.47	0.000	
Russia	0.37	2.35	0.000	
**ASIA**				
China	0.33	2.46	0.000	
Hong Kong	0.56	2.13	0.000	
India	0.14	2.42	0.001	
Indonesia	0.30	2.50	0.000	
Malaysia	0.25	2.49	0.001	
Pakistan	0.25	2.45	0.000	
South Korea	0.37	2.35	0.000	
**MIDDLE EAST**				
Iran	0.22	2.25	0.000	
Israel	0.46	2.27	0.000	
Saudi Arabia	0.45	1.96	0.000	
Turkey	0.36	2.38	0.000	
**AFRICA**				
Ghana	0.16	2.55	0.013	
Kenya	0.24	2.50	0.000	
Nigeria	0.11	2.54	0.000	
Uganda	0.26	2.41	0.000	
**MIDDLE AND SOUTH AMERICA**				
Brazil	0.27	2.56	0.000	
Mexico	0.29	2.51	0.000	

*N = 7973; All p-values are two-tailed. GDP in $1000; ($\bar{DC}$_j_) = nation-level mean of dyadic coping; (DC_ij_ − $\bar{DC}$_j_) = difference between each individual's dyadic coping from their nation-level mean*.

#### Hypothesis 1b

**Figure 2:**
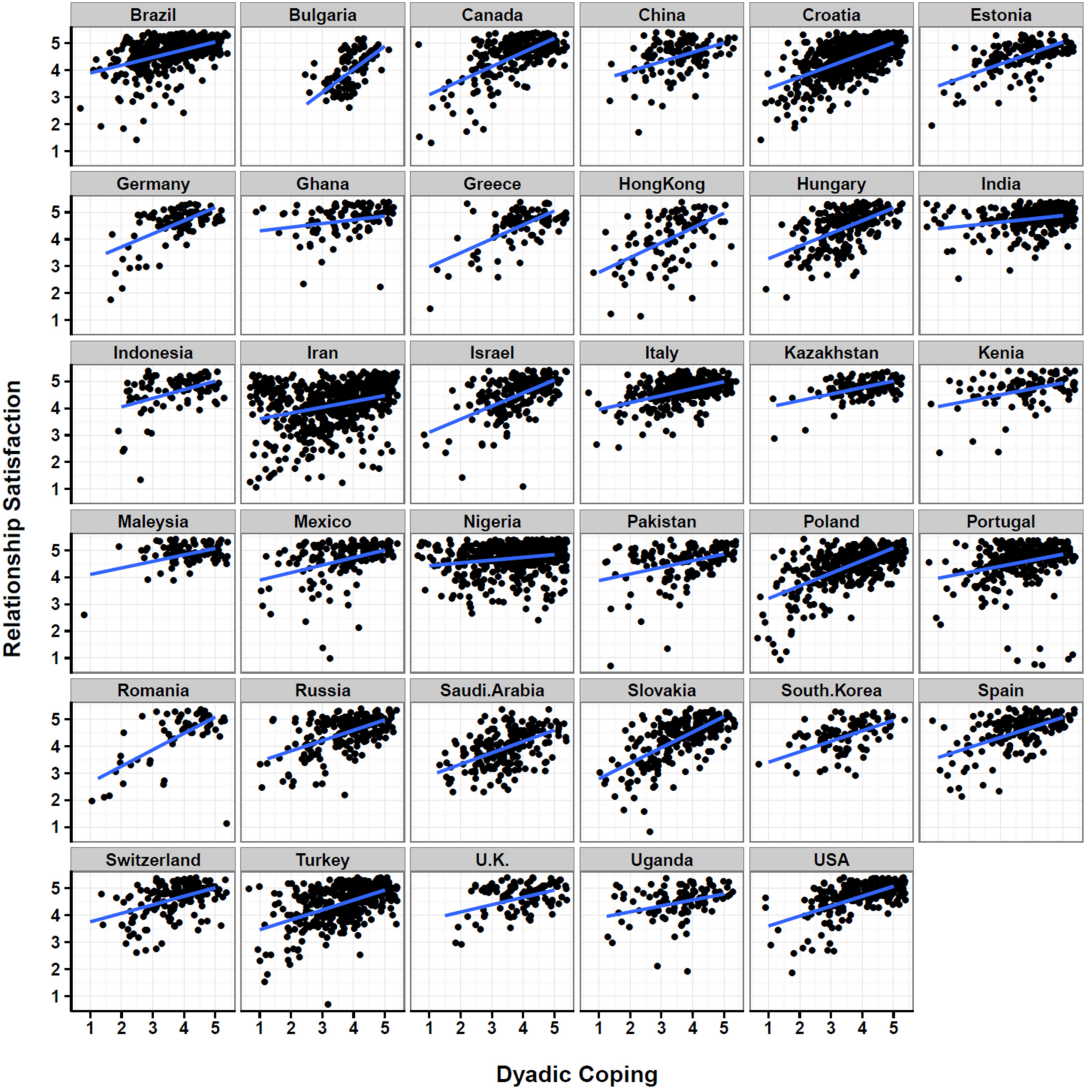
**Scatterplot matrix between dyadic coping and relationship satisfaction for all participants across all nations with nation specific average linear fitting line**.

We hypothesized that the association between dyadic coping (*DC*_ij_ –${\bar{DC}}_{\text{j}}$) and marital satisfaction were significant in each nation as well as that there was significant variability in the slopes between the individual nations (i.e., random effects). Figure 2 shows the association for participants within each nation with an average linear fitting line. The visual inspection supports the overall assumption of positive associations between dyadic coping and marital satisfaction across nations, but it also highlights variably across nations.

Random effects are presented in the lower panel of Table 2. Results show that the associations between dyadic coping and relationship satisfaction vary across nations. The highest association was found in Bulgaria (β = 0.78; *p* < 0.001) and the lowest in Nigeria (β = 0.11; *p* < 0.001). In addition, we computed a *post-hoc* analysis (LR chi-square test) to examine whether the variability in the random effects was significant by comparing a model with the random effects to a model without random effects. Results show that the model with random effects fits the data better [chi-square (2) = 404, *p* < 0.001], indicating significant variability across countries.

**Figure 3:**
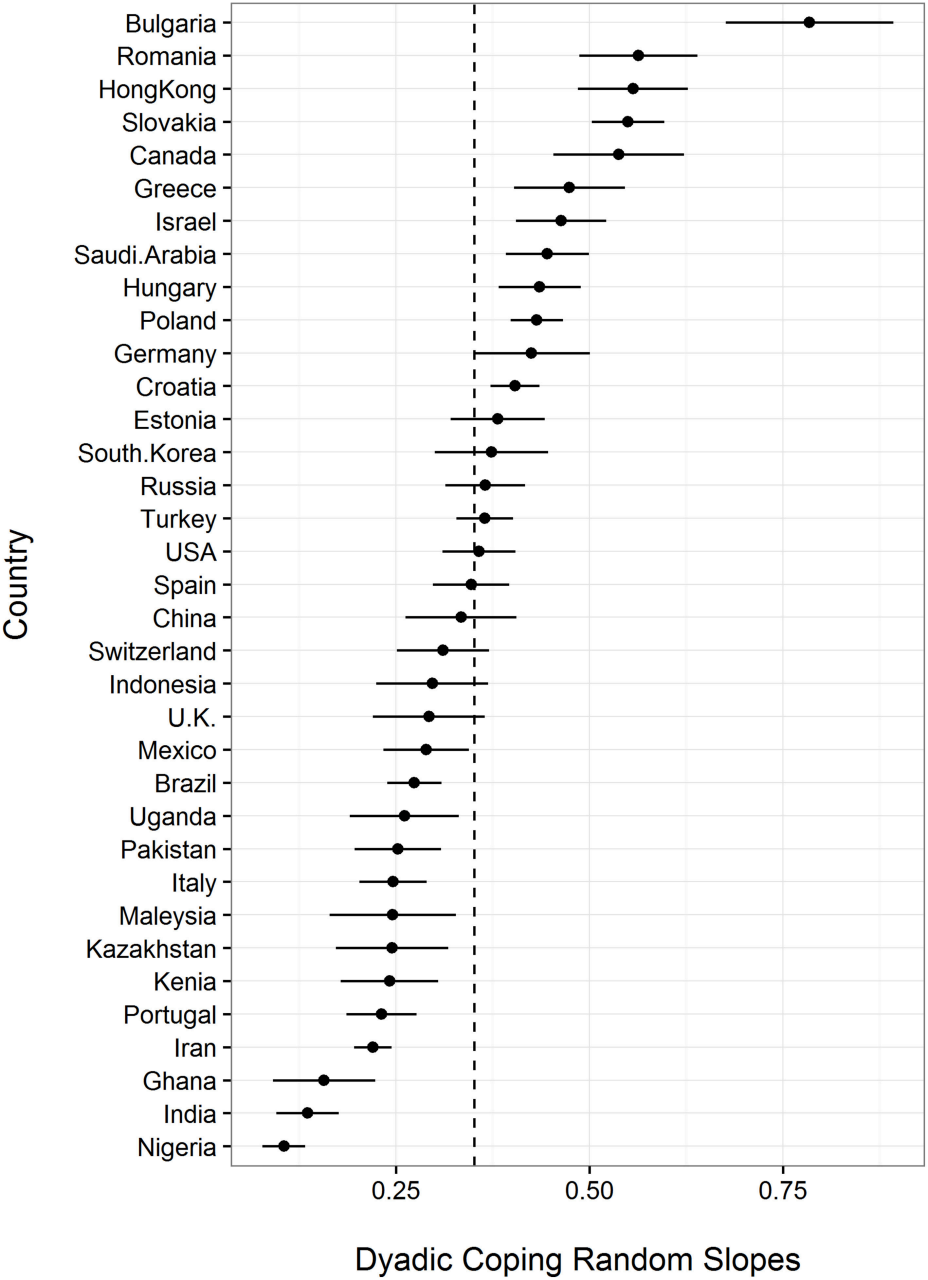
**Random slopes for all nations, ranked according to their strength**.

Furthermore, we tested whether the associations between dyadic coping and relationship satisfaction differed between nations using a graphical approach. In Figure 3, the random slopes are depicted, ranked according to the strength of predicting marital satisfaction. In line with our hypotheses, the visual inspection shows that the confidence intervals do not overlap for all nations, which suggests significant differences (Goldstein and Healy, 1995).

Although we have no specific hypotheses about nations in which the slope (i.e., the association between dyadic coping and relationship satisfaction) would be higher or lower, we use an exploratory approach to compare slopes between some of the nations. Table 2 shows all random slopes for each nation clustered in larger geographical areas. Figure 3 shows random slope estimates with confidence intervals, where intervals that do not overlap across nations indicate statistically significant differences at *p* < 0.05 between them (Goldstein and Healy, 1995). As nearly all previous studies about dyadic coping were examined in North America and West Europe (Falconier et al., 2015), we use these nations as a reference group. Results show the slope for Canada and Germany are significantly higher than for Italy and Portugal, indicating significant difference between these nations. Comparing East European nations, result shows that the slope for Bulgaria was significantly higher than all other nations. Although this might be an outlier, all other slopes for the East European nations were clearly above the average slope of β = 0.35, indicating that for all these nations dyadic coping has a stronger effect on marital satisfaction than for most other nations. For the former Soviet nations, we found slopes that were close to the sample average. For the nations in Asia we found relatively large variabilities. The effect of dyadic coping on relationship satisfaction was significantly higher in Hong Kong than in any other Asian nations. Most slopes were in the range of the population average, expect for India where the slope was significantly lower than for the other Asian nations. In nations from the Middle East, we found relatively high effects for Israel, Saudi Arabia, and Turkey, but a significantly lower slope for Iran. The African nations differed significantly in their slopes. However, the African nations were below the average slope of β = 0.35 suggesting the dyadic coping has a less strong effect on relationship satisfaction in African couples. Finally, slopes for Brazil and Mexico for similar to the average slope of β = 0.35 across all nations.

#### Hypothesis 2

**Figure 4:**
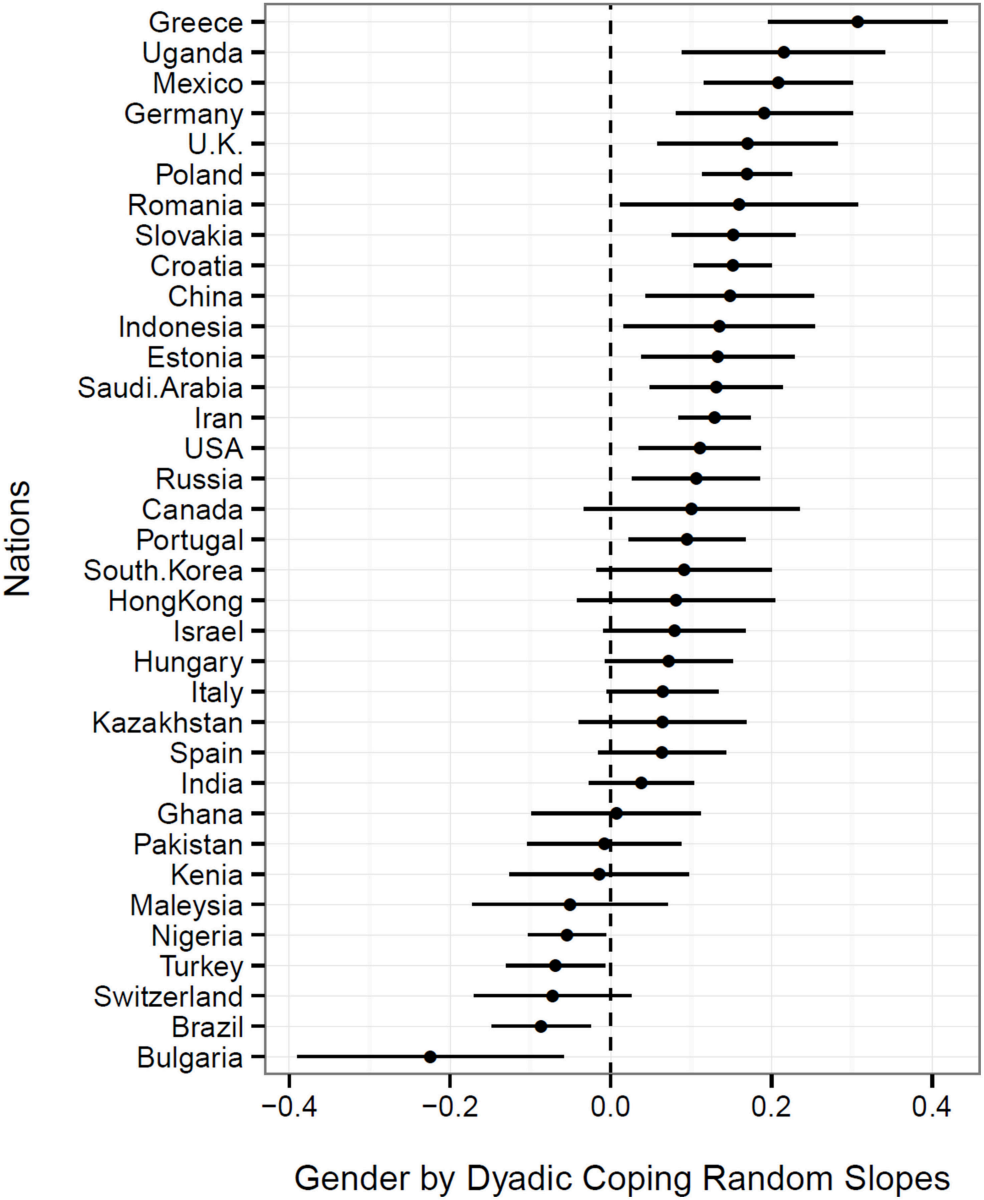
**Random interaction slopes between gender and dyadic coping predicting relationship satisfaction**. Random interaction slopes are significant if the confidence interval does not cross the zero line. If the random interaction slopes are on the left side, the effect is stronger for men whereas when the random interaction slope is on the right side the effect is stronger for women.

Finally, we hypothesized that the association between dyadic coping and relationship satisfaction to be different for husbands and wives (fixed effect) and within nations (random effects). In line with our hypotheses, results showed that gender moderated the association of dyadic coping with relationship satisfaction significantly (fixed effect; β = 0.07; *p* < 0.001), which indicates that, on average, the association between dyadic coping and relationship satisfaction is stronger for women. We used a graphical representation to test whether the moderation effect of gender is different for specific nations (Figure 4). If the confidence interval is crossing zero, there is no significant gender effect (Goldstein and Healy, 1995). The results of the moderation effect of gender show that men benefit more from dyadic coping in 4 nations, whereas the effect of dyadic coping was stronger for women in 17 nations, and no gender effect was found in the remaining 14 nations.

## Discussion

A plethora of research suggests that culture can influence human behavior in general (Markus and Kitayama, 1991; Triandis, 2001); but less is known about how culture affects behavior in intimate relationships. According to the STM (Bodenmann, 1995, 2005), one's cultural contextual environment—family structure, gender roles, and communication—may influence how couples cope with stress in their relationship (Falconier et al., 2016). However, a recent meta-analysis shows that the association between dyadic coping and relationship satisfaction does not vary significantly by nationality or gender (for a meta-analysis see Falconier et al., 2015). Dyadic coping and subsequent effects on individual and relational well-being have been almost exclusively examined in Western couples living in North America and West Europe. The goal of the present study was to address this gap in the literature by testing the theoretical assumption of cultural influence on intimate relationship processes by examining the associations between dyadic coping and relationship satisfaction for married individual across 35 nations.

### Mean differences across nations

Descriptive statistics showed that there are mean differences in how much partners report received dyadic coping and in how satisfied couples are with their marriage across nations. For example, couples in Africa seem to perceive more dyadic coping from their partner and are more satisfied with their relationship, whereas couples in Hong Kong and South Korea report to exchange less dyadic coping behavior and are less satisfied with the marriage. Overall, these findings support conceptual models predicting that the coping process in couples is influenced by culture (Revenson, 1990, 2003; Bodenmann, 1995; Berg and Upchurch, 2007). In addition, results not only highlight difference between nations but also large variability of coping behavior and relationship satisfaction within nations. In summary, these findings indicate that there is variability in the frequency of dyadic coping behavior and marital satisfaction across nations but also within nations. In the following we examine if the *associations* between dyadic coping and marital satisfaction varies between- and within-nations.

### Dyadic coping between nations and the within-nations average (H1a)

Our hypotheses are built around the assumption that partner's coping behavior predicts marital satisfaction. The association of dyadic coping between nations was a significant predictor of relationship satisfaction across all 35 nations (β = 0.59), suggesting that participants are more satisfied with their marriage on average in nations where those participants report to receive more dyadic coping in comparison with participants from nations reporting to receive less dyadic coping. Furthermore, we computed the association between dyadic coping behavior and relationship satisfaction for each nation (i.e., couples share the same cultural environment within a specific nation). The result of the average within-nation association indicates that partners who perceive more dyadic coping by their partner reported more marital satisfaction in comparison with partners who perceive less dyadic coping, which is supported by prior research (Revenson, 1990, 2003; Bodenmann, 1995; Berg and Upchurch, 2007). However, it is noteworthy that the average within-nation effect was smaller than what was found in the recent meta-analysis Falconier and colleagues (β = 0.45; Falconier et al., 2015), which was based almost exclusively on Western samples. Thus, our results provide an initial indication that the association between dyadic coping and relationship satisfaction may vary across nations if examined globally.

### Dyadic coping within nations (H1b)

We hypothesized that the effect of dyadic coping on marital satisfaction (i.e., within-nations slopes) would vary between nations. Results show that the within-nation associations between coping and marital satisfaction vary significantly between nations. This finding not only confirms the conceptual assumption that cultural influences the coping process in couples (Revenson, 1990, 2003; Bodenmann, 1995; Berg and Upchurch, 2007) but it provides evidence that the same amount of coping behavior affects couples across nations differently. For example, coping provision in couples from Hong Kong is associated with a strong increase in marital satisfaction, whereas the same coping provision in couples from Ghana or Kenya has only a small positive impact on the relationship. Notably, we find nations where the frequency of provided coping behavior is high and the coping behavior has a strong impact on the relationship (e.g., Bulgaria, Canada, Greek); but we also find nations where coping behavior is frequently provided but the effect on the relationship is small (e.g., Ghana, Kenya, Nigeria); or where coping behavior is relatively rare but the impact on the relationship is (e.g., Hong Kong, Saudi Arabia). This is a crucial finding as it provides new insights into the coping process in couples. Thus far, we assumed that partners benefit when providing more coping behavior to each other, which is also a basic idea of relationship education programs such as Couples Coping Enhancement Training (Bodenmann and Shantinath, 2004; Bodenmann et al., 2014) and Couple CARE (Halford et al., 2004). However, our findings indicate that the impact of dyadic coping is influenced by the culture couples live in. This highlights that couple relationship education programs need to be tested and potentially adapted before implementing them in non-Western cultures, as the effect of a specific skill training (e.g., training of dyadic coping) might not lead to the same effect on relationship outcomes across cultures in comparison with the effect found for programs with Western couples (Bodenmann and Shantinath, 2004; Halford et al., 2004; Bodenmann et al., 2014).

Although we did not have specific hypotheses for individual countries, we did an exploratory examination of countries by region. We first examined whether the random slopes differed across nations. This allowed us to use the findings as a baseline and compare them to findings in other regions. Nations in North American and West Europe showed significant differences between their within-nation slopes. We found higher random slopes for Canada and Germany in comparison with U.K., Italy, and Portugal. This suggests that cultural differences seem to affect the association between dyadic coping and relationship satisfaction even in this relatively homogeneous Western cultures. These also stands in sharp contrast to the findings of the meta-analysis by Falconier et al. (2015), which found no differences in the effect of dyadic coping on relationships across Western nations.

Notably, we found hardly any difference in the association between dyadic coping and marital satisfaction across larger regions in comparison with the average association in North America and West Europe, but we found significant variability between nations clustered in a larger region. In more detail, the slopes in regions such as the former Soviet nations, Middle East, Asia, and Middle- and South America fluctuate around the average slope (β = 0.35)—and the association of dyadic coping on marital satisfaction is comparable with the average effect in North America and Western Europe. Surprisingly, however, we also found significant differences between nations in most of the larger regions, which could indicate that the associations between dyadic coping and relationship satisfaction in areas such as North American, West European, former Soviet, Middle East, Asian, and Middle- and South American nations are similar. This is notable, as one could expect differences across these regions based on cultural differences. In addition, we found that nations differ significantly between each other within specific areas. This means that in regions where we expect that cultures are relatively similar we find significant differences between specific nations (e.g., Canada and U.S., Spain and Portugal, Slovakia and Hungary, Hong Kong and China, Pakistan and India, Saudi Arabia and Iran). Future research should examine causes why the effect of dyadic coping varies across cultural similar nations more than expected.

Finally, we found that nations from Eastern Europe and Africa were different than our comparison region of North America and Western Europe. On average, the slopes from Eastern Europe were significantly higher than the average slope of β = 0.35. Specifically, we found the highest slope for Bulgaria, but because the data from Bulgaria was unique in other ways (low Cronbach alpha for dyadic coping; lowest level of relationship satisfaction) this should be interpreted with caution (i.e., outlier). But even if we do not take Bulgaria into consideration, the random slopes in all these Eastern European nations were much higher than the average in any other region. Although all the slopes were high in the nations in this region, we still found significant differences between nations in this region (e.g., higher values for Romania and Slovakia; lower values for Poland and Croatia). For the African nations in our sample, we found that their slopes were lower than in any other larger region such as North America and Western Europe, indicating that dyadic coping seems to be less related to relationship satisfaction for couples living in Africa. However, comparing between nations we found higher random slopes in Ghana, Kenya, and Uganda in comparisons with the slope in Nigeria.

### Control variables

In order to make the non-stratified sample more comparable, we included a variety of control variables. Results show no association between individualism-collectivism and marital satisfaction. Inspecting the slopes in Figure 3, we could not find any specific pattern based on individualism-collectivism. Rather, the strongest and the weakest slopes were found in collectivistic cultures, indicating that the association between dyadic coping and marital satisfaction cannot be predicted by individualism-collectivism. Results further revealed that women, younger participants and more educated participants are more satisfied with their marriage in comparison with men, older participants and less educated participants respectively. Finally, results also indicate that couples are more satisfied in nations with higher GDPs in comparison with couples living in nations with lower GDPs, which might indicate that couples in more wealthy nations face less stressors.

## Gender as moderator

### H2a

Results provide evidence for our hypothesis that the association between dyadic coping and relationship is different for husbands and wives across all nations, which is in sharp contrast to the meta-analysis findings (Falconier et al., 2015). This contrast indicates that culture is a crucial factor to predict gender differences in coping processes in couples. These processes can be better examined if we study these effects for each individual nation individually.

### H2b

Based on theoretical assumptions (e.g., evolutionary perspective, attachment theory; Bolby, 1969) and cultural differences (e.g., gender role; Eagly and Wood, 1999), we further hypothesized that the association between dyadic coping and relationship satisfaction is higher for women than for men, at least in some nations. The results of the random effects show significant gender interactions for 60% of all nations. In contrast to our assumption, we found four nations where the associations were stronger for men (Brazil, Bulgaria, Nigeria, Turkey). These results contradict our theoretical assumption that the effect of dyadic coping is either stronger for women or there should be no difference between genders, but findings this effect just in 4 of 35 nations indicates that he in general the association is in general stronger for women or equal across genders. In line with our assumption, we found that in 17 nations the effect of dyadic coping on relationship satisfaction was stronger for women and in 14 nations we found no interaction with gender. Thus, finding a stronger association for some but not all nations provides some support for cultural specific causes. Based on the findings of the meta-analysis (Falconier et al., 2015), we did not expect to find significant interactions with gender for nations in North America or West Europe. However, the association between dyadic coping and marital satisfaction was higher for women in several Western nations (Greece, Germany, Poland, U.K., Croatia, Slovakia, Portugal, and USA). Future studies should examine why we find gender differences in some Western cultures but not in others.

## Strengths and limitations

The confidence in our results is supported by several strengths. First, this study is based on a large sample of married individuals (*N* = 7973) from 35 different nations. Gathering such a large sample allowed us to address the gap in the literature about the associations between culture and couples' coping processes, which affords researchers greater specificity on the influence of culture in the coping process of couples. Second, our analysis account for nested data (i.e., individuals nested in specific nations) which is important because if nested data are not modeled accordingly it violates statistical assumption of independence between individuals (Eid and Lischetzke, 2013). Third, our analysis differentiated between and within nation components of dyadic coping, which allowed us to examine between- and within-nation effects. Finally, it is in general difficult to test within nation effects for 35 nations against each other, as this yields 595 comparisons. The graphical approach allows the reader to visually inspect whether the association between dyadic coping and relationship is significantly different between any nations.

Despite these strengths, several limitations should be noted. First, the data collected are based on convenience samples, which limits the generalizability. Furthermore, the recruitment strategies may have differed across nations; however, we controlled for several important aspects (e.g., education, marital duration, number of children) to allow us to make the results more comparable across nations. Nevertheless, future research should look at stratified samples to make findings between nations more comparable. Second, different cultural groups might interpret items differently based on cultural characteristic (Schwartz et al., 2014) which might contribute to the variability in our findings. However, the current approach to test for measurement invariance (multi-group confirmatory factor analysis) is suitable in cases when just few groups are compared—but should not use testing for measurement invariance across 35 nations (Muthen and Asparouhov, 2013); furthermore the maximum-likelihood approach assumes fully invariant parameters, which is too restrict for a heterogeneous sample such as ours (Muthen and Asparouhov, 2013). Third, data was collected from individuals, rather than data from both partners within the relationship, which does not allow us to examine processes between bother members of a couple. As the data was collected from married heterosexuals, the results may not be generalizable to unmarried individuals (cohabitating or not) in opposite- or same-sex relationships. Fourth, the analysis relies on cross-sectional data, which limits conclusions about causality in the data. Finally, we have limited data from South America and do not have data from all countries, which limits our generalizations for all nations. Despite these limitations, our data is based on 7973 married participants from 35 nations living in 4 continents, which gives insight into the variability of the associations between dyadic coping and relationship satisfaction in couples.

## Implications and future directions

Culture, undoubtedly, impacts the association between dyadic coping behavior and relationship satisfaction (Revenson, 1990, 2003; Bodenmann, 1995; Berg and Upchurch, 2007). This might be even more important as standardized couple programs train skills like dyadic coping behavior (CCET, Bodenmann et al., 2014, p. 2014; Bodenmann and Shantinath, 2004), but our findings provide evidence that the impact of such behavioral skills varies across nations. While there are couple interventions that can help couples in troubled relationships (Halford et al., 2010; Bodenmann et al., 2014), their effectiveness across cultures has yet to be determined. Theoretically, while couple's prevention and intervention programs could be beneficial for all couples, it is crucial to examine whether a skill, such as dyadic coping which has shown to have cross-cultural benefits (Falconier et al., 2016), can be taught to all couples. Finally, the current study focuses mainly on between-person differences—those people are more satisfied with their marriage who perceive more support from their partner. But it is reasonable to assume that coping processes fluctuate within-persons over time. Therefore, future research should use daily diary methods to examine how situation-specific processes affect coping process in couples.

## Author contributions

PH contribution: Initial research design, data collection and analysis, manuscript drafting, and final approval. AR contribution: Data collection and analysis, manuscript drafting, and final approval. PS contribution: Initial research design, data collection and analysis, manuscript drafting, and final approval. DA contribution: Initial research design, data collection, manuscript drafting, and final approval. AS contribution: Initial research design, data collection, and final approval. All other co-authors' contribution: Data collection, manuscript corrections, and final approval.

## Funding

PS and AS were supported by funds of Polish Ministry of Science and Higher Education (scholarships to PS for years 2012–2017, and scholarship to AS for years 2013–2016). ES applied for funding for data collection, which was awarded by the Public Welfare Foundation Propondis. All other studies were based on individual contributions.

### Conflict of interest statement

The authors declare that the research was conducted in the absence of any commercial or financial relationships that could be construed as a potential conflict of interest.

## Footnotes

^1^Retrieved September 16, 2015, from https://www.r-project.org/

^2^We computed a random intercept model and found significant variance for the random intercepts across nations in dyadic coping (95% CI [0.13, 0.23]) and for marital satisfaction (95% CI [0.17, 0.27]).

^3^We also tested control variables for significant differences across countries (one-way ANOVA). Because even small differences will be significant based on the large sample size, we computed effects sizes across all nations and average effect size across all nations for age (*d*_average_ = 0.51), marital duration (*d*_average_ = 0.51), amount of children (*d*_average_ = 0.45), and education level (*d*_average_ = 0.46) indicating some variabilities across nations.
