# Corrigendum: The Associations of Dyadic Coping and Relationship Satisfaction Vary between and within Nations: A 35-Nation Study

**DOI:** 10.3389/fpsyg.2016.01404

**Published:** 2016-09-27

**Authors:** Peter Hilpert, Ashley K. Randall, Piotr Sorokowski, David C. Atkins, Agnieszka Sorokowska, Khodabakhsh Ahmadi, Ahmad M. Alghraibeh, Richmond Aryeetey, Anna Bertoni, Karim Bettache, Marta Błażejewska, Guy Bodenmann, Jessica Borders, Tiago S. Bortolini, Marina Butovskaya, Felipe N. Castro, Hakan Cetinkaya, Diana Cunha, Oana A. David, Anita DeLongis, Fahd A. Dileym, Alejandra D. C. Domínguez Espinosa, Silvia Donato, Daria Dronova, Seda Dural, Maryanne Fisher, Tomasz Frackowiak, Evrim Gulbetekin, Aslıhan Hamamcıoğlu Akkaya, Karolina Hansen, Wallisen T. Hattori, Ivana Hromatko, Raffaella Iafrate, Bawo O. James, Feng Jiang, Charles O. Kimamo, David B. King, Fırat Koç, Amos Laar, Fívia De Araújo Lopes, Rocio Martinez, Norbert Mesko, Natalya Molodovskaya, Khadijeh Moradi, Zahrasadat Motahari, Jean C. Natividade, Joseph Ntayi, Oluyinka Ojedokun, Mohd S. B. Omar-Fauzee, Ike E. Onyishi, Barış Özener, Anna Paluszak, Alda Portugal, Ana P. Relvas, Muhammad Rizwan, Svjetlana Salkičević, Ivan Sarmány-Schuller, Eftychia Stamkou, Stanislava Stoyanova, Denisa Šukolová, Nina Sutresna, Meri Tadinac, Andero Teras, Edna L. Tinoco Ponciano, Ritu Tripathi, Nachiketa Tripathi, Mamta Tripathi, Noa Vilchinsky, Feng Xu, Maria E. Yamamoto, Gyesook Yoo

**Affiliations:** ^1^Department of Psychiatry and Behavioral Sciences, University of WashingtonSeattle, DC, USA; ^2^Department of Psychology, University of ZurichZurich, Switzerland; ^3^Counseling and Counseling Psychology, Arizona State UniversityTempe, AZ, USA; ^4^Institute of Psychology, University of WroclawWroclaw, Poland; ^5^Behavioral Sciences Research Center, Baqiyatallah University of Medical SciencesTehran, Iran; ^6^Department of Psychology, College of Education, King Saud UniversityRiyadh, Saudi Arabia; ^7^School of Public Health, University of GhanaLegon, Ghana; ^8^Department of Psychology, Catholic University of MilanMilan, Italy; ^9^Department of Psychology, The Chinese University of Hong KongHong Kong, China; ^10^Graduate Program in Morphological Sciences, Federal University of Rio de JaneiroRio de Janeiro, Brazil; ^11^Cognitive and Behavioral Neuroscience Unit, D'Or Institute for Research and EducationRio de Janeiro, Brazil; ^12^Institute of Ethnology and Anthropology, Russian Academy of SciencesMoscow, Russia; ^13^Laboratory of Evolution of Human Behavior, Federal University of Rio Grande do NorteNatal, Brazil; ^14^Department of Psychology, Faculty of Languages History and Geography, Ankara UniversityAnkara, Turkey; ^15^Faculty of Psychology and Education Sciences, University of CoimbraCoimbra, Portugal; ^16^Department of Clinical Psychology and Psychotherapy, Babes-Bolyai University Cluj-NapocaCluj-Napoca, Romania; ^17^Department of Psychology, University of British ColumbiaVancouver, BC, Canada; ^18^Department of Psychology, King Saud UniversityRiyadh, Saudi Arabia; ^19^Department of Psychology, Universidad IberoamericanaCiudad de Mexico, Mexico; ^20^Faculty of Arts and Sciences, Izmir University of EconomicsIzmir, Turkey; ^21^Department of Psychology, Saint Mary's UniversityHalifax, NS, Canada; ^22^Department of Psychology, Akdeniz UniversityAntalya, Turkey; ^23^Department of Anthropology, Cumhuriyet UniversitySivas, Turkey; ^24^Faculty of Psychology, University of WarsawWarsaw, Poland; ^25^Department of Public Health, Medical School, Federal University of UberlândiaUberlândia, Brazil; ^26^Department of Psychology, University of ZagrebZagreb, Croatia; ^27^Department of Clinical Services, Federal Neuro-Psychiatric HospitalBenin-City, Nigeria; ^28^Department of Organization and Human Resources Management, Central University of Finance and EconomicsBeijing, China; ^29^Department of Psychology, University of NairobiNairobi, Kenya; ^30^Department of Psychology, Simon Fraser UniversityBurnaby, BC, Canada; ^31^Department of Anatomy, Baskent UniversityAnkara, Turkey; ^32^Department of Social Psychology, University of GranadaGranada, Spain; ^33^Institute of Psychology, University of PécsPécs, Hungary; ^34^Department of Agricultural Extension and Education, Razi UniversityKermanshah, Iran; ^35^Institute of Psychology, University of Science and CultureTehran, Iran; ^36^Department of Psychology, Pontifical Catholic University of Rio de JaneiroRio de Janeiro, Brazil; ^37^Faculty of Computing and Management Science, Makerere University Business SchoolKampala, Uganda; ^38^Department of Pure & Applied Psychology, Adekunle Ajasin UniversityAkungba-Akoko, Nigeria; ^39^School of Education and Modern Languages, Universiti Utara MalaysiaSintok, Malaysia; ^40^Department of Psychology, University of NigeriaNsukka, Nigeria; ^41^Department of Anthropology, Istanbul UniversityIstanbul, Turkey; ^42^Faculty of Arts and Humanities, University of MadeiraFunchal, Portugal; ^43^Institute of Clinical Psychology, University of KarachiKarachi, Pakistan; ^44^Department of Psychological Sciences, Constantine The Philosopher University in NitraNitra, Slovakia; ^45^Department of Social Psychology, University of AmsterdamAmsterdam, Netherlands; ^46^Department of Psychology, South-West University “Neofit Rilski”Blagoevgrad, Bulgaria; ^47^Department of Psychology, Matej Bel University in Banská BystricaBanská Bystrica, Slovakia; ^48^Faculty of Sports and Health Education, Indonesia University of EducationBandung, Indonesia; ^49^Institute of Psychology, University of TartuTartu, Estonia; ^50^Institute of Psychology, University of the State of Rio de JaneiroRio de Janeiro, Brazil; ^51^Organizational Behaviour and Human Resource Management, Indian Institute of Management BangaloreBangalore, India; ^52^Department of Humanities and Social Sciences, Indian Institute of Technology GuwahatiGuwahati, India; ^53^Department of Psychology, Bar-Ilan UniversityRamat-Gan, Israel; ^54^Department of Education for Students, Guangdong Construction PolytechnicGuangdong, China; ^55^Department of Child & Family Studies, Kyung Hee UniversitySeoul, South Korea

**Keywords:** dyadic coping, relationship satisfaction, culture, multilevel modeling, gender differences

Due to an oversight, the name of the author “Ahmad M. Alghraibeh” was incorrectly spelled as “Ahmad M. Aghraibeh.” The correct version is shown above. The authors apologize for this oversight. This error does not affect the scientific conclusions of the article in any way.

The original article has been updated.

## Conflict of Interest Statement

The authors declare that the research was conducted in the absence of any commercial or financial relationships that could be construed as a potential conflict of interest.

